# Learning manufacturing computer vision systems using tiny YOLOv4

**DOI:** 10.3389/frobt.2024.1331249

**Published:** 2024-06-12

**Authors:** Adan Medina, Russel Bradley, Wenhao Xu, Pedro Ponce, Brian Anthony, Arturo Molina

**Affiliations:** ^1^ School of Engineering and Sciences, Tecnologico de Monterrey, Tlalpan, Mexico; ^2^ Department of Mechanical Engineering, School of Engineering, Massachusetts Institute of Technology, Cambridge, MA, United States; ^3^ Institute of Advanced Materials and Sustainable Manufacturing, Tecnologico de Monterrey, Tlalpan, Mexico

**Keywords:** education, educational innovation, higher education, manufacturing, quality control, object detection, Tiny YOLO v4, computer vision

## Abstract

Implementing and deploying advanced technologies are principal in improving manufacturing processes, signifying a transformative stride in the industrial sector. Computer vision plays a crucial innovation role during this technological advancement, demonstrating broad applicability and profound impact across various industrial operations. This pivotal technology is not merely an additive enhancement but a revolutionary approach that redefines quality control, automation, and operational efficiency parameters in manufacturing landscapes. By integrating computer vision, industries are positioned to optimize their current processes significantly and spearhead innovations that could set new standards for future industrial endeavors. However, the integration of computer vision in these contexts necessitates comprehensive training programs for operators, given this advanced system’s complexity and abstract nature. Historically, training modalities have grappled with the complexities of understanding concepts as advanced as computer vision. Despite these challenges, computer vision has recently surged to the forefront across various disciplines, attributed to its versatility and superior performance, often matching or exceeding the capabilities of other established technologies. Nonetheless, there is a noticeable knowledge gap among students, particularly in comprehending the application of Artificial Intelligence (AI) within Computer Vision. This disconnect underscores the need for an educational paradigm transcending traditional theoretical instruction. Cultivating a more practical understanding of the symbiotic relationship between AI and computer vision is essential. To address this, the current work proposes a project-based instructional approach to bridge the educational divide. This methodology will enable students to engage directly with the practical aspects of computer vision applications within AI. By guiding students through a hands-on project, they will learn how to effectively utilize a dataset, train an object detection model, and implement it within a microcomputer infrastructure. This immersive experience is intended to bolster theoretical knowledge and provide a practical understanding of deploying AI techniques within computer vision. The main goal is to equip students with a robust skill set that translates into practical acumen, preparing a competent workforce to navigate and innovate in the complex landscape of Industry 4.0. This approach emphasizes the criticality of adapting educational strategies to meet the evolving demands of advanced technological infrastructures. It ensures that emerging professionals are adept at harnessing the potential of transformative tools like computer vision in industrial settings.

## 1 Introduction

The imperative of instructing students in complex Artificial Intelligence (AI) implementations has ascended to prominence within contemporary university curriculums. The diverse applications that AI boasts across various fields have rendered it an indispensable inclusion in the curriculum plans of engineering degrees. However, the difficulties inherent in this subject may engender anxiety among students, particularly if they perceive the concepts as impossible (Wang et al., 2022). Therefore, this paper proposes a project-based learning strategy to enhance students’ understanding of the subject matter and mitigate the apprehension it could evoke by facilitating hands-on engagement and real-world application.

Computer Vision (CV) plays a pivotal role in optimizing and organizing the material flow on the production floor, which is crucial for streamlining, enhancing efficiency, and minimizing errors within manufacturing processes. Comprehensive learning about CV’s applications in managing material flow, requires students to understand how automated sorting mechanisms can categorize materials based on distinct characteristics such as size, shape, and color. Such computerized systems negate the need for manual intervention, thereby boosting efficiency and curtailing labor costs. Further, real-time monitoring of material logistics using CV, maintains operational continuity and pinpoints bottlenecks. Simultaneously, the data harnessed from CV systems are instrumental in informed decision-making processes related to material flow and inventory management. The ability of CV to trace materials throughout the manufacturing stages is indispensable for quality assurance, compliance with regulatory standards, and maintaining comprehensive inspection trails. Additionally, CV supports robotic configurations in accurately identifying, selecting, and positioning materials, amplifying precision and velocity in material handling tasks. Understanding the integration of CV into Automated Guided Vehicles (AGVs) and conveyor systems offers insights into the uninterrupted transportation of materials and the overarching automation of material flow on the production floor (Korchagin et al., 2021).

Safety protocols are also substantially bolstered through CV’s capacity to identify potential hazards related to material movement, such as overflow or obstructions, significantly diminishing accident risks. Insights into how CV ensures compliance with safety regulations during material transportation and handling are fundamental in fostering a secure operational atmosphere. CV’s role in minimizing material loss is equally paramount, achieved by identifying and signaling any material discrepancies during transit, thereby preventing operational disruptions stemming from material deficits. Concurrently, the continuous inspection of materials as they progress along the production line guarantees adherence to quality benchmarks. The faculty of CV to discern and isolate substandard materials ensures the manufacturing echelon is preserved. CV systems can be adapted to various materials to accommodate the unique demands of diverse manufacturing environments. Students must comprehend how these systems can be modified to meet differing material and product specifications. By grasping the integral function of Computer Vision in managing material flow within production areas, students acquire competencies that deal with the difficulties and technological strides of the modern manufacturing sector. This understanding enhances their career preparation and gives them the proficiency to drive forward technological innovations (Meier, 2022).

Teaching complicated subjects has regularly been a challenging endeavor. Students exhibit diverse learning modalities regardless of the educator’s proficiency, particularly in assimilating abstract concepts. Scholars advocate for more experiential teaching strategies like project-based learning. However, Williams suggested that relying solely on a practical project, devoid of foundational theoretical instruction, can be counterproductive. Nevertheless, it is often more advisable than a curriculum that is entirely theory-laden (Williams, 2003).

Integrating artificial intelligence (AI) into education indicates a transformative era, offering unparalleled opportunities to improve various disciplines. A critical aspect lies in equipping undergraduate students with the proficiency to comprehend and implement cutting-edge technologies in their respective fields. This necessitates establishing a robust framework tailored to student projects, underscoring its importance. Numerous scholars have advocated for pedagogical approaches to imbue undergraduates with AI proficiency. For instance (Eaton, 2017), advocates for a project-centric curriculum employing robots to acquaint students with the fusion of AI Computer Vision (CV) and advanced robotics. Similarly, Murphy (1998) encourage a course catering to chemistry students, facilitating their utilization of object detection techniques. Despite their efficacy, these methodologies are often confined within specific domains, posing challenges in their broader application across diverse fields. A critical observation reveals that existing literature predominantly focuses on integrating AI tools into educational curricula, neglecting the imperative of fostering students’ capability to conceptualize and execute AI projects that facilitate learning. Consequently, a notable absence of methodologies is dedicated to teaching AI in CV for manufacturing applications, compounded by the absence of a universal framework adaptable to varied disciplines. In light of these deficiencies, the imperative to devise a comprehensive framework for student-led projects becomes evident. Such a framework empowers students to delve into AI applications and cultivates their problem-solving and critical-thinking skills. Students gain firsthand experience tackling real-world challenges by embarking on project-based learning journeys, bridging the gap between theoretical knowledge and practical implementation. Creating a versatile framework for student project development is an indispensable endeavor in modern education. Beyond imparting technical competencies, it fosters innovation and interdisciplinary collaboration and prepares the next-generation of professionals to navigate the intricacies of AI integration across diverse domains.

**FIGURE 1:**
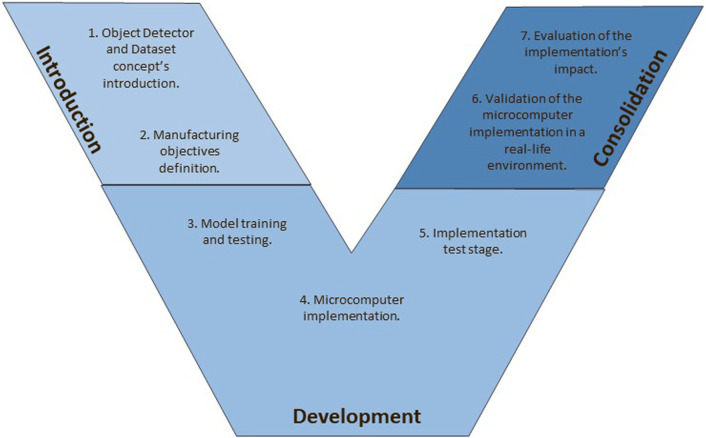
V-model competences methodology.

AI shows an abstract domain encompassing a vast array of applications across multiple fields, presenting a daunting task for educators aiming to convey these tools’ extensive capabilities comprehensively. A meticulously designed project can explain to students the pragmatic applications of AI, particularly in specialized fields such as CV. CV, being more visual, can aid in students’ understanding of the concepts since the correctness of the CV algorithm is apparent just by looking at the output of the model. Moreover, it is paramount for students to discern the extensive applicability of these concepts in various practical arenas, including manufacturing. Consequently, this work adopts a manufacturing project as the medium for instruction. The planning of this project holds a V-model-based methodological framework (Ponce et al., 2021), delineated in Figure 1. This structured approach initiates with an introductory overview provided by the educator, explaining the mechanics of the chosen object detection algorithm. Subsequently, students are shown through the network training process, an integral phase that marks the culmination of the introductory competencies segment. Upon the training’s completion, the model undergoes rigorous testing to ascertain its readiness for practical deployment.

The V-model, initially developed in the late 1980s for software development, represents a structured approach that illustrates the sequential nature of the development process and its corresponding testing stages. Its primary appeal is explicitly depicting the relationships between different development lifecycle phases, facilitating a thorough understanding and management of the process. Over the years, the V-model has been adapted and optimized to cater to the evolving needs of various technological domains beyond its original software-centric application. This adaptability has led to its application in developing Cyber-Physical Systems (CPS), integrations of computation, networking, and physical processes. The model has been tailored to address the unique challenges of CPS, ensuring a systematic and disciplined approach to their development (Gräßler et al., 2021). Additionally, the V-model is relevant in electrical and electronic product development. In this context, the model supports the intricate process of designing and implementing complex electronic systems, helping to streamline the development process and enhance efficiency (Bauer et al., 2022). Moreover, the V-model has been effectively employed in specific manufacturing contexts, particularly in Additive Manufacturing (AM). In AM processes, the V-model provides a comprehensive framework for managing the development of new products or technologies. It ensures that each stage of the development process is systematically planned and executed, leading to the successful implementation of AM projects (De Lima et al., 2023). In summary, with its structured and phased approach, the V-model has proven to be a versatile and robust framework that extends well beyond its original software development context. Its ability to clearly define and link the various stages of the development process has made it an enduring and adaptable tool relevant to multiple technological and manufacturing advancements.

The ensuing stages encompass comprehensive testing and a comprehensive evaluation of the model’s real-world performance, signifying the transition from the development phase to the consolidation of competencies. This systematic process solidifies students’ understanding and immerses them in a practical scenario, fostering a holistic learning experience. The final phase, comprising a detailed assessment of the entire implementation, marks the peak of the competency consolidation stage.

This didactic strategy, emphasizing experiential learning through a blend of foundational theory and hands-on application, is poised to enhance students’ cognitive engagement, thereby deepening their understanding of AI’s multifaceted role in contemporary domains like CV. Furthermore, this approach equips them with the expertise to navigate and contribute significantly to the ever-evolving technological landscape.

The manufacturing sector is specifically selected for this educational framework, acknowledging the substantial likelihood of students pursuing professional avenues within this industry post-graduation. Hence, immersing them in this context provides a pragmatic learning experience directly translatable to their future occupational settings. Moreover, manufacturing is a dynamic field characterized by constant evolution, with practitioners and researchers striving to integrate cutting-edge innovations to augment efficiency across diverse facets of the industry.

Deep Learning in computer vision is one of the most versatile and adaptable tools in this continuous advancement, due to its capabilities to handle large amounts of data and process it very fast and have the capability of learning from the data to adapt to scenarios (Russell and Norvig, 2010). Its efficacy hinges on the capacity to train the neural network with a robust dataset, empowering it to surmount numerous limitations that conventional computer vision methodologies encounter. Such advanced capabilities facilitate the implementation of these networks in various segments of the manufacturing chain, surpassing the traditional confines that more straightforward computer vision tools typically inhabit.

A distinctive advantage of employing more sophisticated algorithms instead of essential solutions is their adaptability in challenging environments. These advanced systems maintain their performance integrity even under conditions marred by occlusions, suboptimal lighting, or lower-resolution imagery. This resilience is attributable to the training phase, wherein the model is exposed to various images captured in diverse settings. The system acquires a comprehensive experiential base by including these variations, enhancing its interpretative accuracy and operational adaptability.

Thus, the learning process prepares students for real-world industrial challenges and introduces a deep understanding of the transformative potential inherent in advanced AI technologies like Deep Learning-based computer vision. This knowledge is instrumental in navigating the present complexities and contributing innovatively to the future landscape of an ever-evolving manufacturing sector.

This work introduces a versatile framework designed to facilitate the teaching of complex subjects. It uses artificial intelligence (AI) applied in computer vision (CV) for manufacturing as an illustrative example. The core aim is to equip undergraduate students with the skills to apply various AI tools in real-world scenarios, transcending the specific domain of AI in CV. The framework is intended to be adaptable across different fields, ensuring that students can transfer and apply the learned concepts to diverse professional settings. The proposed framework integrates theoretical and practical elements. It begins with a simplified classroom approach that introduces general concepts, avoiding intricate details. This theoretical foundation is linked to the broader research landscape, highlighting many ideas and tools that can be leveraged in practical applications. The emphasis is on facilitating students’ engagement with a specific research topic they can implement in a project. This project-oriented approach encourages students to internalize general principles and apply them to devise solutions in their respective fields. To validate the efficacy of this framework, a combination of theoretical examinations and student-initiated projects is proposed. These assessments gauge students’ ability to translate theoretical knowledge into practical, field-specific applications.

**FIGURE 2:**
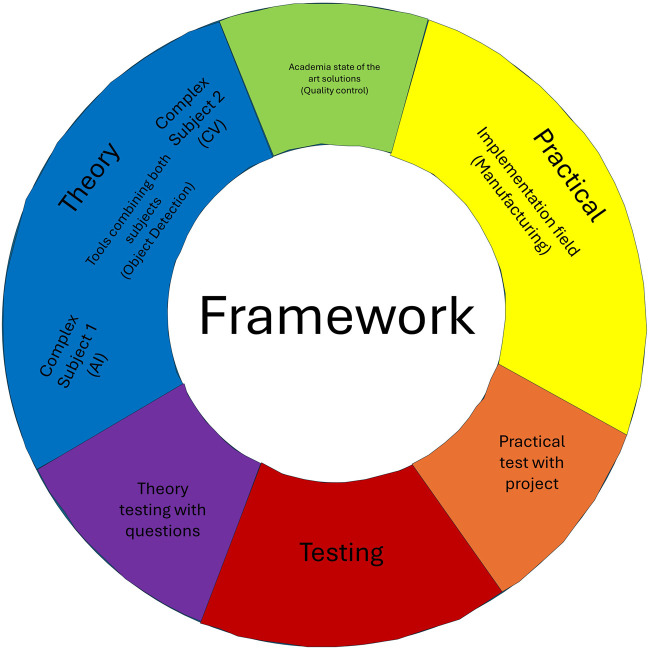
Proposed framework.

Figure 2 provides a schematic of the framework, illustrating the integration of research areas—specifically AI and CV—and their application in a practical context like manufacturing. However, the framework’s flexibility allows these elements to be tailored to other sectors, such as agriculture, where AI could be used for tasks like detecting plant diseases or identifying ripe fruits for robotic harvesting. The proposed framework is designed to be a dynamic teaching tool that covers the specifics of AI and CV and empowers students to apply these technologies across various domains. It fosters a comprehensive understanding and innovative application of theoretical concepts in practical scenarios.

## 2 Computer vision project based on manufacturing

There are a lot of new solutions to be found with the integration of complex subjects like AI and CV. Object detectors are flexible algorithms that allow machines to sense the world in a way that is closer to the humans perception, which helps researchers implement these algorithms more easily, this can be seen in the different implementations that has been presented in the research world to solve common problems in different fields, such as agriculture to implement these solutions in harvesting robots (Wang C. et al., 2023), or energy efficiency by using object detectors to more accurately detect clothing insulation for thermal comfort calculation in HVAC use (Medina et al., 2022a); autonomous driving has also tried these algorithms to detect pedestrians (Chen and Lin, 2019), other cars (Jin et al., 2020) and even traffic signals (Medina et al., 2022b). Therefore, the importance of learning how and when to implement these algorithms is no longer something left for the graduate students and researchers alone, undergraduate students must know these tools to help them implement in their workplace, and help these different fields evolve.

Since there is a high percentage of engineering students that will work in the manufacturing industry, a manufacturing project is presented as an example in this paper.

### 2.1 Theoretical framework

This proposal imparts a theoretical framework that shows certain concepts, subsequently allowing students to witness the tangible outcomes following implementation. Nonetheless, it is crucial to underscore the significance of these implementations within manufacturing environments to students. Additionally, illustrating alternative techniques is fundamental to fostering comparative analytical thinking. Manufacturing is a behemoth industry, historically at the forefront of spearheading myriad technical innovations, particularly in automating production lines. However, the uncompromising emphasis on product quality remains central to these manufacturing processes. Regardless of the procedures’ sophistication, sustaining a consistent quality standard is paramount. Consequently, quality control has become one of the most rigorously studied and indispensable facets of manufacturing.

Computer Vision (CV) integrates various technological instruments designed for rapid, non-intrusive product inspections, positioning it as an attractive industry solution. These tools have garnered acclaim for their straightforward operation, rapidity, and unparalleled precision. Their integration into manufacturing practices marks a transformative shift, and here we outline several vital applications that demonstrate the extent of CV’s influence (Zhou et al., 2022).

#### 2.1.1 Fault detection

Computer Vision systems are trained to detect product abnormalities or defects, ensuring that only high-quality items are shipped. This aids in reducing wastage and enhancing customer satisfaction by preventing defective products from reaching the market.

#### 2.1.2 Inventory management

Computer Vision helps track stock levels precisely, recognizing products and their quantities, which results in efficient inventory management. It reduces manpower and time needed while minimizing errors associated with manual inventory tracking and ensures real-time, accurate data availability.

#### 2.1.3 Safety monitoring

It enables monitoring of workers to ensure adherence to safety protocols, detecting whether safety equipment like helmets and gloves are being appropriately used, and identifying potential hazardous situations to prevent accidents.

#### 2.1.4 Quality control

Beyond defect detection, Computer Vision aids in analyzing product quality across manufacturing stages, ensuring that each product adheres to the set quality standards and specifications.

#### 2.1.5 Predictive maintenance

Computer Vision identifies wear and tear in machinery, predicting when maintenance is due or when a breakdown is imminent. This leads to reduced downtime and increases operational efficiency.

#### 2.1.6 Robotic guidance

It is used for guiding robots on the assembly line, enabling them to interact with objects and execute tasks like picking, placing, welding, and assembling precisely and quickly.

#### 2.1.7 Assembly verification

Computer Vision verifies whether components are assembled correctly in real time, ensuring the integrity of the manufacturing process and reducing errors in production lines.

#### 2.1.8 Product classification and sorting

It automates product classification and sorting based on characteristics such as size, shape, and color, increasing throughput and efficiency.

#### 2.1.9 Dimensional accuracy

Computer Vision assesses the dimensional accuracy of products by measuring dimensions in real time to ensure they meet the specified tolerances, enhancing product reliability.

#### 2.1.10 Material handling

With Computer Vision, systems can identify, pick, and place materials accurately and efficiently, optimizing the material handling process in manufacturing.

#### 2.1.11 No-entry zone supervision

Computer Vision helps monitor restricted areas within the manufacturing unit and ensures that unauthorized personnel do not enter these zones, thereby maintaining safety.

#### 2.1.12 Optical character recognition (OCR)

OCR in Computer Vision is used to read, extract, and authenticate printed or handwritten text information, aiding in tasks like reading labels, expiration dates, and batch numbers.

Each application of Computer Vision plays a central role in enhancing manufacturing processes by minimizing errors, fortifying safety protocols, and bolstering overall productivity within the sector. Among the array of techniques, some have already found commercial applications, including color detection. Color detection is crucial for product classification and establishing item dispositions based on color. More than mere sorting, this method verifies that products are infused with the intended colors. Examples span a variety of products, from soda and paint cans to fabrics, labels, pharmaceutical capsules, and sample books. Implementing this technique requires a color sensor with precise calibration and strategically positioned cameras or color sensors within a rigorously controlled lighting environment. The label manufacturing industry heavily relies on this technique, given the paramount significance of color accuracy for labels, another important manufacturing industry that relies heavily on color sensing for quality control is wire manufacturing (Tsai and Cheng, 2022).

**FIGURE 3:**
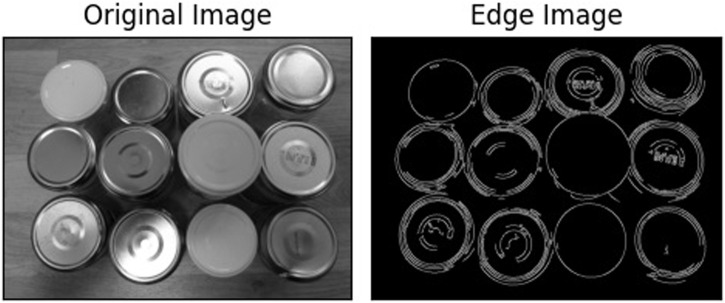
Edge detection algorithm.

Edge detection is another sophisticated computer vision technique crucial for isolating desired objects within an image (Prince, 2012). This process involves initially identifying the target object and then eliminating background information to prevent miscalculations in edge definition. Given their acute sensitivity to lighting conditions and the contrast between objects and their surroundings, these procedures demand accurate execution. A filter—often Gaussian—is first employed to mitigate noise (Bradski and Kaehler, 2008). The image is then converted to a binary format, intensifying the contrast essential for differentiating the object from its background and isolating the target information. Establishing a threshold is critical in discerning what is identified as white and what is relegated as black, with the flexibility to tweak this demarcation to enhance precision. Subsequently, pixel gradients and associated directions are calculated, identifying areas of sudden color transitions. Although viable with color images, this method proves most potent with transitions from black to white or vice versa. For color imagery, thresholds are applicable, yet binary conversions often provide superior accuracy. The Canny edge detector is a typical algorithm for this purpose, renowned for its precision in discerning edges. Figure 3 illustrates an example of this advanced algorithm at work. At work, this example is created using the can lid dataset obtained from Kaggle at https://www.kaggle.com/code/rrighart/detection-of-product-defects-using-yolov7/input. By understanding these complex techniques, stakeholders in manufacturing can harness Computer Vision’s full potential, optimizing processes and outcomes in this ever-evolving industrial landscape.

### 2.2 Dataset and object detection concept’s introduction

A more advanced solution for quality control involves integrating Artificial Intelligence (AI) techniques with computer vision systems. The adaptability inherent in such integration enables smooth translation across various settings, with correctly trained AI proving less susceptible to variables like lighting conditions, camera angles, occlusions, or low-resolution imaging. Significantly, the versatility of AI extends beyond computer vision, as demonstrated by numerous studies showcasing diverse industrial applications of these algorithms (Lee et al., 2017). Compiling a comprehensive dataset is central to successfully implementing this sophisticated technique. This dataset can be curated from custom images, those sourced from the internet, or collections from platforms offering datasets for purchase or free. While paid datasets usually undergo rigorous error-checking and feature a wide variety of scenarios, they can be costly, mainly if there is a need to amalgamate multiple datasets to encompass a range of object instances. In contrast, free datasets, although financially more accessible, may contain mislabeling and lack diversity in background scenarios, potentially resulting in suboptimal feature extraction and false detections. The selected images must be rich in background detail for effective model training. Utilizing images with uniform color or plain backgrounds could impede the model’s capability in feature recognition, as training for image classification often relies on such images, centering on single objects amidst minimal background interference. Optimally, images should blend objects with their surroundings and even include occlusions to enhance the model’s proficiency in object localization during training sessions, this can either be intentionally captured when building the dataset or artificially made using mosaic transformations (Hao and Zhili, 2020). Once a robust collection of images is collected, the training phase of object detectors should emphasize both the classification and precise localization of objects. This precision underscores the importance of including images with occlusions in the dataset. Following this, the crucial process of labeling or annotating the dataset begins. Object detection involves drawing bounding boxes around the objects and categorizing them.

For the annotation process, complimentary tools are available, such as labelImg (Tzutalin, 2015), accessible via its GitHub repository. This user-friendly tool facilitates the meticulous drawing of bounding boxes around the objects, ensuring they do not intersect with object edges and omit irrelevant background content. Moreover, labelImg is versatile, offering label generation in compatible formats and detailing each bounding box’s class, center coordinates, width, and height, streamlining the annotation process for enhanced model training (Tzutalin, 2015).

**FIGURE 4:**
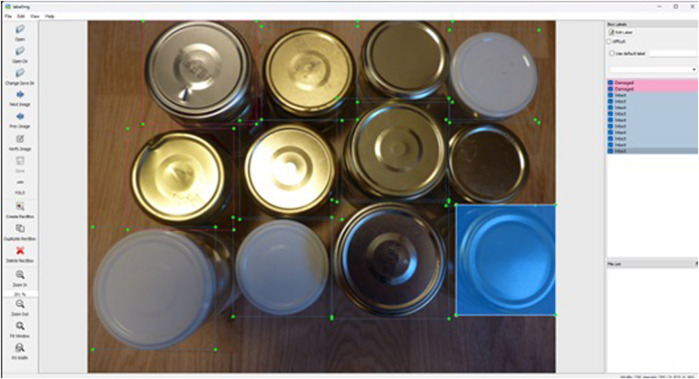
LabelImg tool.

Figure 4 visually represents the labelImg tool’s functionalities, showcasing various features such as the ability to open individual images or an entire directory allocated for training and testing. Additionally, the tool offers navigation controls to peruse images and select labeling formats, with the You Only Look Once (YOLO) format, which corresponds to an algorithm that will be discussed later, being the choice in this example. The interface includes creating and duplicating rectangles, deleting, and zooming options. On the right side of the screen, the labels assigned to each bounding box are visible, allowing for modifying a selected bounding box label, which is accentuated in blue in the visual and dark blue within the label list. Furthermore, Figure 4 details the labeling process, with green indicators marking the corners of each bounding box, each tied to a specific label. The image’s left segment displays a toolbox, enabling users to craft additional bounding boxes via the “Create Rectangle” feature. This action modifies the cursor into an extended cross along the *X* and *Y*-axes, simplifying the labeling process for objects with non-linear perimeters. After establishing the bounding box through a left-click and drag motion, a dialog box appears for label input.

**FIGURE 5:**
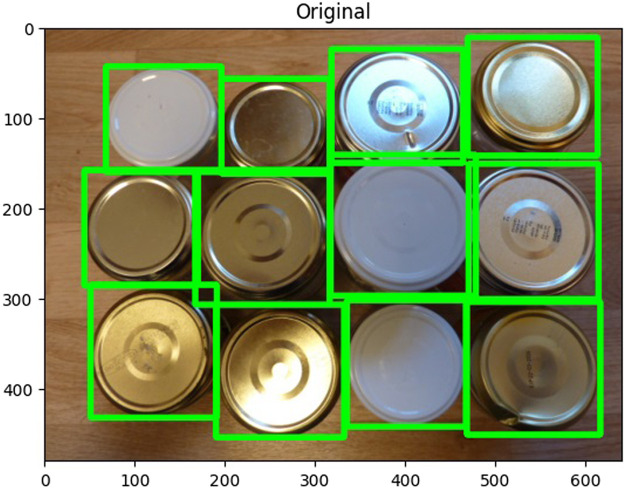
Original image.

**FIGURE 6:**
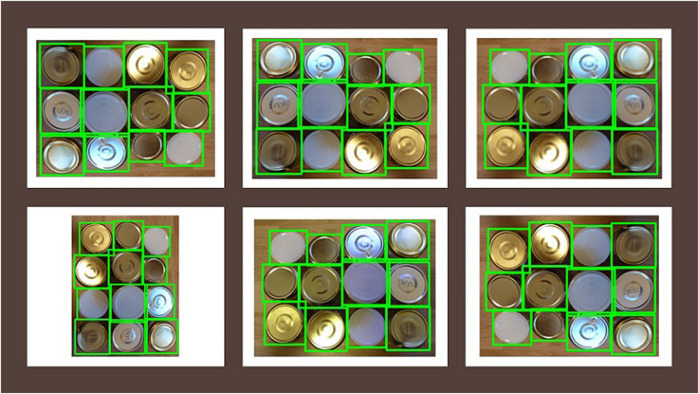
Vertical flip (top left), horizontal flip (middle top), average blur (top right), rotation (bottom left), vertical and horizontal flip (middle bottom) and raise hue (bottom right).

Data augmentation becomes valuable when datasets are scarce or limited to a few hundred images. This approach enhances the size of the image dataset by applying various computer vision methods to alter existing images. Data augmentation is particularly beneficial when time restrictions prevent the collection of an ample dataset. Transformations applied include adjustments to characteristics such as contrast, brightness, color, orientation, and the introduction of noise or blur. These adaptations expand the dataset and prepare the model for training in diverse conditions, including suboptimal lighting or reduced image resolution. One innovative method involves cropping sections of objects and assembling collages, thereby generating occlusions that compel the object detector to identify objects within more intricate environments. Figure 5 exhibits the initial image selected for modification, while Figure 6 illustrates a series of transformations. These altered images are produced using CLODSA (Casado-García et al., 2019), a Python-based application that not only adjusts images but also adapts bounding boxes as needed—for instance, when an image flip requires a repositioning of the original coordinates to maintain alignment with the bounding box, assuming it is perfectly centered.

Numerous object detection algorithms exist, but many researchers agree that among the most efficient lightweight models are the YOLO algorithms (Redmon et al., 2016). These algorithms are particularly advantageous because they are open source, providing a GitHub repository that users can clone, thereby facilitating the training of custom models. Notably, the scaled-down versions of these algorithms, often referred to as “Tiny” versions or “n” in more recent iterations, have proven fast and accurate enough for use on constrained systems like the Raspberry Pi 4. The training time for these algorithms can vary depending on the number of classes and images, and it differs across various implementations. While there are no strict guidelines, a common practice is to train the model for approximately 2,000 epochs per class, ensuring that the total number of epochs is at least equal to the number of images. Object detection has shown significant effectiveness in manufacturing environments, serving roles beyond merely identifying defective items. It is crucial in recognizing safety equipment on personnel, monitoring restricted areas for unauthorized access, and more. Numerous potential applications remain unexplored, underscoring the adaptability of these models. However, it is vital to keep similar problems within the scope of a single algorithm to maintain the integrity of the model’s performance. For example, a model designed for detecting defective items should not be mixed with functionality for identifying safety equipment. The strength of these models lies in their versatility, allowing them to address a wide range of issues within certain limitations. By focusing on solving related problems within one model, we can maximize the benefits of object detection across various manufacturing contexts, ensuring superior outcomes.

As stated in the introduction, a project-based approach can facilitate a better understanding of complex concepts, though a certain degree of theoretical background is still necessary. This foundation helps students grasp the subject matter more comprehensively and prevents potential misconceptions or misinterpretations during project execution. Therefore, a brief overview of the YOLO algorithm is provided to deepen readers’ comprehension of its workings. It is essential to recognize that specific details may differ between versions; hence, it is paramount to confirm the version in use and comprehend these differences.

**FIGURE 7:**
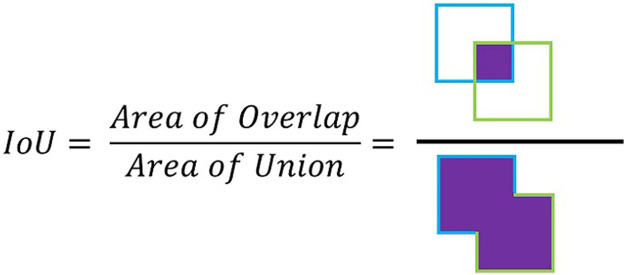
Intersection over Union (IoU).

YOLOv4 begins by resizing the input image to a 640 × 640 pixel resolution, a task accomplished by the OpenCV library. The image is then introduced into the convolutional neural network, which undergoes various operations and is eventually converted into a matrix. This matrix represents every pixel’s information throughout the color channels. YOLOv4 (Bochkovskiy et al., 2020) employs pre-established boxes called anchor boxes for object detection. These boxes have predetermined height and width dimensions that mirror the training bounding boxes, but they differ in shape and position to cover various object forms throughout the image. Each anchor box is assigned a score that reflects the likelihood of an object’s presence within it. The system then adjusts the center’s location and the bounding box’s width and height based on the offset between the anchor box and the training bounding box. This offset is measured using the Intersection over Union (IoU) metric, representing the overlapping area’s ratio between the predicted bounding box and the original bounding boxes to the total area encompassing both, as illustrated in Figure 7.

**FIGURE 8:**
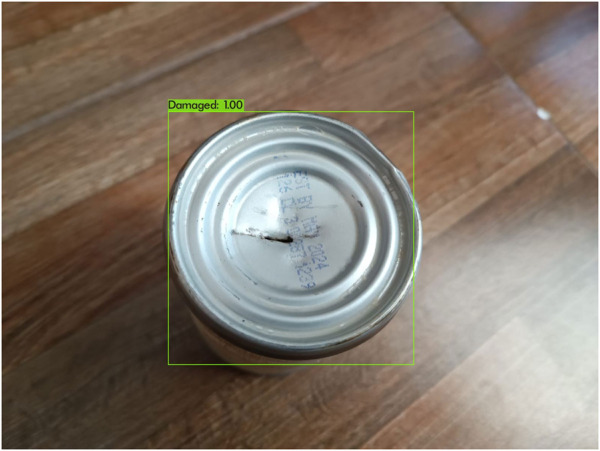
Bounding box surrounding a damaged can with the correct class “Damaged” and the probability “1.00” shown.

The YOLO algorithm notably enables object localization by drawing bounding boxes around identified objects. This can be seen in Figure 8, which is a result of the trained algorithm. You can see how a rectangle is drawn around the detected object; this rectangle is known as container box. And then the class and probability that the detected object belongs to that class, according to the algorithm, placed on the top left of the container box, the probability is given as a floating-point value between 0 and 1 where 1 is equivalent to 100%.

The classification phase is an integral component of the algorithm, during which the system generates a probability score reflecting the likelihood that the detected object belongs to a specific class. This score is derived from the features extracted from within the bounding box. To finalize the creation of a bounding box in the output, a threshold value is applied to filter out detected objects associated with lower probabilities, distinguishing them from those confidently recognized as the targeted objects. Typically, this threshold is set at 0.5, equating to a 50% probability.

**FIGURE 9:**
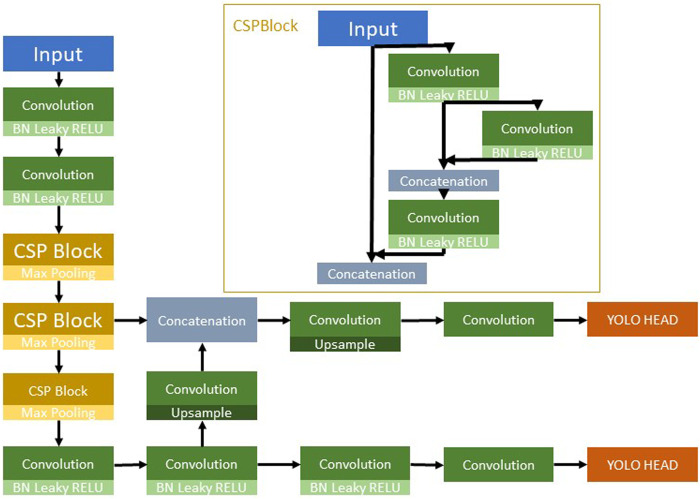
YOLOv4 Tiny architecture.

A distinctive feature of the YOLO architecture is its ability to conduct detections and classifications at different scales: three in its full versions and two in the “Tiny” iterations. This multi-scale approach enhances the algorithm’s ability to detect objects of various sizes within an image. YOLO also boasts custom feature extractors, termed “backbones,” which form the convolutional neural network architecture responsible for pulling out pertinent features required for object classification and detection. For instance, YOLOv4 employs a backbone known as CSPDarknet53, which draws inspiration from the DenseNet architecture. In contrast, TinyYOLOv4 uses a more compact variant named CSPDarknet53Tiny; this can be seen in Figure 9, which depicts the YOLOv4 Tiny architecture (Jiang et al., 2020). These backbones are pivotal in the network’s learning process, significantly influencing its performance and the accuracy of the features it extracts for subsequent classification and detection tasks.

Newer models such as YOLOv5 (Jocher et al., 2022), YOLOv6 (Li et al., 2022), YOLOv7 (Wang C. Y. et al., 2023), YOLOv8 (Jocher et al., 2024) and YOLOv9 (Wang et al., 2024), changed the size of the possible algorithms to include more options, YOLOv5 for example, include five different algorithm sizes ranging from the smallest named YOLOv5n, often referred to as nano, to the largest which is YOLOv5x. This size difference is done by scaling different algorithm parameters to change the algorithm’s size. Since the same authors developed YOLOv8, it shares the exact five different sizes. YOLOv6 only has four different sizes, and YOLOv7 has six different sizes, while the most recent one, YOLOv9, has only five different sizes. Another significant change at the beginning of this model is the migration from the Darknet framework developed by the original creator of YOLO Redmon to a more general framework named PyTorch. The main difference between these two frameworks is that Darknet is more restrictive with system compatibilities; since it was developed in a Linux environment, Linux operating systems have straightforward installation procedures. However, other operating systems have more steps and complications to install. On the other hand, PyTorch is more friendly with different operating systems and can be more easily transferred to different operating systems. Another difference is that the YOLOv5 was built towards more efficient training by noticing when the model stops learning and preventing overfitting.

The evolution of the YOLO (You Only Look Once) algorithm has seen various iterations, each bringing forward unique optimizations that enhance its performance in object detection. While it is possible to discuss the entire architecture of each version, the focus here will be on notable improvements that have significantly influenced the algorithm’s development and application. Starting from YOLOv6, a distinct shift was made from the traditional anchor-based detection method to an anchor-free detector. This transition marks a pivotal change as the model no longer relies on offset values to adjust the center coordinates of bounding boxes. Instead, it directly proposes these coordinates, streamlining the process and reducing computational complexity, leading to faster inference times. In the subsequent version, YOLOv7, the enhancement was primarily in the feature extraction phase of the algorithm. The authors introduced a novel convergence technique, Extended ELAN (Exponential Linear Activation Network), designed to expedite the model’s convergence by optimizing the gradient path, thus facilitating a more efficient learning process. The development continued with YOLOv8, which aimed to accelerate the processing speed inherited from YOLOv5 and integrated advancements from YOLOv6, like the anchor-free detection, to refine its performance further.

Moving to YOLOv9, significant strides were made in optimizing feature extraction, drawing from the advancements in YOLOv7. It employed GELAN (Generalized ELAN), an improvement on the previous ELAN, to enhance overall performance. Moreover, YOLOv9 introduced the Programmable Gradient Information (PGI), a technique designed to elevate the efficiency of smaller models, enabling them to achieve performance comparable to larger architectures.

These iterative enhancements in the YOLO series demonstrate the algorithm’s evolutionary trajectory and highlight the absence of peer-reviewed documentation, especially for versions like YOLOv5 and YOLOv8. The latter versions were developed by private entities without published detailed research papers, creating a gap in the formal academic discourse regarding their specific optimizations and technical foundations. Each iteration of the YOLO algorithm represents a step forward in the field of object detection, offering nuanced improvements that address both the computational efficiency and the accuracy of the detection process.

### 2.3 Model training and testing

Students will be provided with a curated image dataset, already labeled, it must be noted that labeling the images can help the student to understand the process however it is encouraged as an exercise for a class to try on a couple of images instead of a huge dataset due to the time consumption it takes; and access to a Google Colab notebook. This notebook will serve as a comprehensive guide, detailing each step necessary to set up the environment and train the TinyYOLOv4 network. Additionally, it will illustrate how students can execute object detection tasks on both image and video files directly within the same notebook. However, it is essential to note that due to the inherent limitations of Google Colab’s cloud-based infrastructure, access to real-time hardware components, such as a camera for live feed detection, is unavailable. Therefore, live detections will require an alternative setup or platform. The instructional notebook includes detailed procedures to clone the repository of the selected algorithm from GitHub, which hosts the essential files needed for this project. It further guides students through creating and configuring the necessary settings to train the detection model using the provided labeled dataset.

**FIGURE 10:**
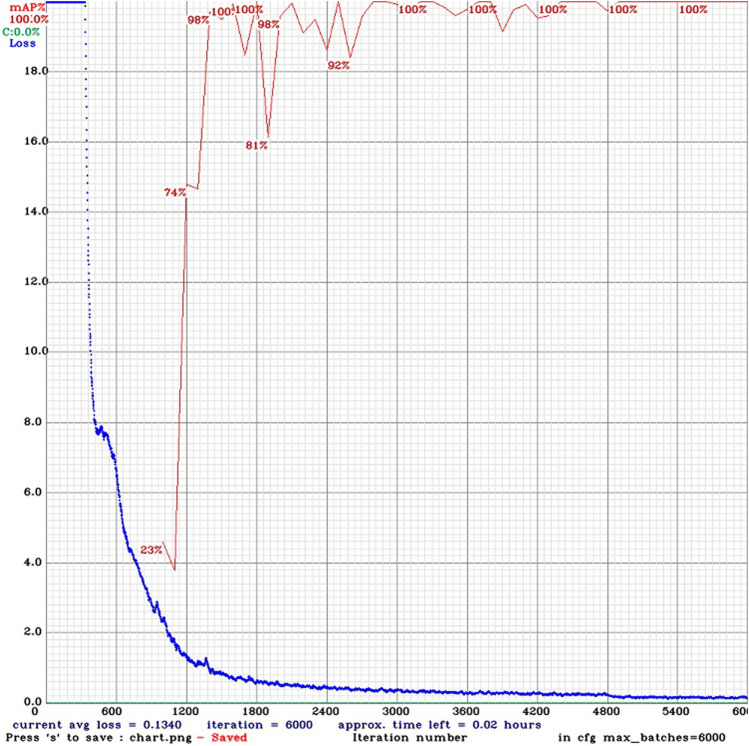
Training results.

Figure 10 shows the results of the training after 6,000 epochs, where the red line indicates the mean Average Precision for the training dataset and the blue line represents the Loss function value.

Moreover, the notebook offers practical examples of how to test the newly trained model by running detection tasks on sample images and videos. Students will learn not only how to initiate these tests but also how to visualize the results within the notebook interface. The notebook demonstrates how students can save their results as a new video file for video testing, allowing them to choose a preferred filename for this output. The entire process, from the initial setup to the training phase, is designed to be efficient and user-friendly. Given that a relatively small data set is utilized for training purposes, the entire compilation of the notebook, including the time taken for the actual training, should not exceed 3 h. This streamlined approach ensures that students can effectively engage with and understand the fundamental aspects of implementing and operating the TinyYOLOv4 within a manageable timeframe.

### 2.4 Implementation

Regarding the object detection algorithm, the next step is its practical implementation. Typically, any Personal Computer (PC) equipped with a GPU can efficiently execute object detection tasks. However, PCs can sometimes restrict system flexibility due to limitations, making embedded implementations more desirable. Embedded systems, in this regard, provide enhanced flexibility in algorithm deployment. However, the specific setup required for hardware computations must be considered. Despite their compact size, devices like the Raspberry Pi 4 can manage these computational demands.

The Raspberry Pi 4 with 8 GB of RAM is chosen for several reasons. Since its creation it was envisioned as an educational tool. This microcomputer has been highly welcomed by students and hobbyists alike, therefore the amount of community support is very big, so if the student wants to use this in another project he does not need a class to make it. Among microcomputers it has one of the best processing units, alongside with a small GPU and 8 GB of RAM making it very flexible for multiple applications. Finally even though it is not the cheapest microcomputer out on the market, it is the best cost-benefit relation considering all the information and projects available, plus its hardware specs.

The Raspberry Pi is a small but impressively powerful computer offering various digital opportunities (Kölling, 2016). It boasts a suite of features that deliver a full-fledged computing experience. Its processing capabilities, encompassing a robust CPU and dedicated GPU, ensure seamless user interaction and high-definition visual content. The device offers multiple RAM options and uses microSD cards for storage, allowing efficient multitasking and adaptable storage solutions. In connectivity, the Raspberry Pi has wireless LAN, Bluetooth, and Ethernet ports, ensuring solid and diverse internet and device connections. It also houses various ports, such as USB for peripherals, HDMI for display output, and General-Purpose Input/Output (GPIO) pins for broad electronic interfacing and Internet of Things (IoT) explorations. The device extends its versatility with dedicated interfaces for cameras and displays. The Camera Serial Interface (CSI) facilitates the addition of a Raspberry Pi camera, which is essential for image and video capture tasks, as well as allowing USB cameras to work with the Raspberry Pi. At the same time, the Display Serial Interface (DSI) accommodates touchscreen displays, enhancing interactive applications. For audio-visual needs, the Raspberry Pi is equipped with a 3.5 mm audio jack and HDMI ports, supporting high-definition video playback, making it suitable for multimedia endeavors. Depending on the model, the power supply is conveniently managed through a USB-C or micro-USB port. Enhancing Raspberry Pi’s user experience is a wealth of documentation and an active community offering ample resources, guidance, and support. Its official operating system, Raspberry Pi OS, includes a range of applications for general and developmental use. Given its compactness and versatility, the Raspberry Pi is an excellent platform for various applications, from educational projects to professional setups. Its extensive features and supportive community make it an invaluable tool in digital exploration. Considering these advantages, the Raspberry Pi is selected for this project’s implementation. Running the object detection algorithm on a live feed can be accomplished using a custom Python script. OpenCV facilitates model recognition through the “readNetFromDarknet” function within the “dnn” module, requiring the network configuration and weights files. The “videoCapture” function captures each frame, which is then processed by the model for detections. Subsequently, bounding boxes are drawn at specific coordinates with the most probable class label displayed alongside, aiding in object classification. This step involves the “NMSBoxes” function, which requires input of predicted boxes and confidence levels. This function outputs a vector detailing each detection, including bounding box coordinates, dimensions, class identity, and associated confidence levels. To refine the results, you can adjust threshold values for object detection and confidence probabilities, ensuring more accurate identifications.

The steps to implement the trained network in the Raspberry Pi are listed below:

1. Make sure that the Raspberry Pi has access to the full SD card space by altering the filesystem configuration and rebooting the Raspberry Pi.
2. Download the configuration file with the. cfg extension and the best weights obtained, and place them in a designated folder inside the Raspberry Pi.
3. Create a Python script that uses opencv mentioned packages to read the network from the configuration file and load up the weights; this script should be in the same folder as the. cfg and weights files.
4. An installation of OpenCV is required however, the version that is for Raspberry Pi is not compatible with the required modules so a virtual environment must be created, changing some lines inside the bashrc file.
5. After initializing the virtual environment in the command prompt, run the script to the folder containing the files using the cd command.

### 2.5 Testing and evaluation

To complete the project, students are tasked with testing the object detection algorithm on a microcomputer, utilizing a webcam to acquire live feed data. Following this practical engagement, they are to conceptualize an application within a manufacturing context, akin to a conveyor belt or batch quality control system, reflecting real-world industrial mechanisms. Additionally, students are expected to articulate insights on potential enhancements to the model’s precision and operational applications.

This less structured project segment gives students the creative autonomy to incorporate elements from previous undertakings, such as automation projects, into their current work. For instance, they might need to engineer a holder for the Raspberry Pi, which accommodates an electrical connector, should they decide on a continuous power supply solution that the implementation context permits. Alternatively, they could explore battery-powered setups to grant the system greater autonomy, broadening the scope of feasible applications. The essential hardware components for this exercise include the Raspberry Pi and a camera. The choice of camera is not restricted, though it is pertinent to note that, as previously mentioned, the algorithm will resize any images to a 640 × 640 resolution. Consequently, higher resolutions will undergo a reduction to align with the algorithm’s parameters. To facilitate real-time monitoring of the detection process, students can employ a display supplemented by a keyboard and mouse for seamless navigation within the Raspberry Pi’s interface. This setup will aid in the initiation of the system and the acquisition of pertinent results.

**FIGURE 11:**
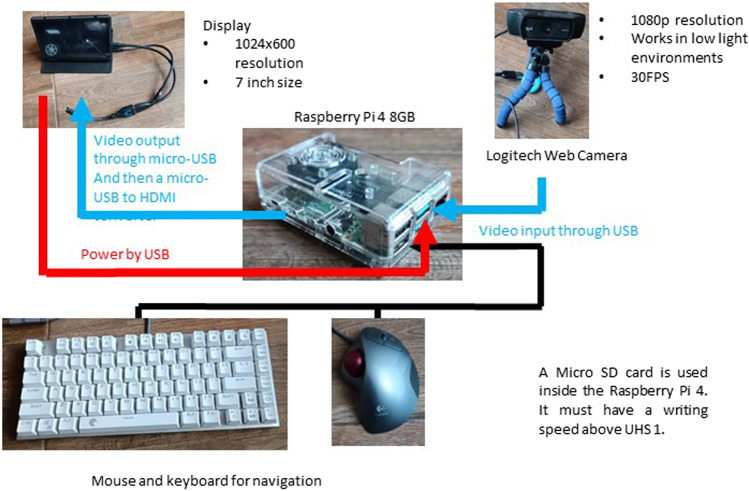
Raspberry Pi deployment.

Figure 11 illustrates the system’s configuration, featuring the Raspberry Pi and a schematic of the connection layout. It is important to highlight that the system is designed to operate via USB connections, negating the need for specialized connectors. This simplicity ensures a user-friendly experience and supports a straightforward replication of the setup, encouraging students to focus more on the innovative aspects of their implementations. By engaging in this project, students are not only applying theoretical knowledge. Still, they are encouraged to think critically and innovatively, proposing improvements and potentially pioneering advancements in object detection applications within industrial settings. This holistic approach aims to provide a comprehensive educational experience, merging foundational learning with practical skill development in a contemporary context.

## 3 Proof of concept

This research aims to create a framework, demonstrated through a proof of concept delivered to students of various majors and academic years in a 2-h session. This session was designed as an optional course, open for registration on short notice, to assess the framework’s educational efficacy rather than provide an exhaustive educational implementation.

The class began with an announcement about its objectives, followed by a pre-session test to establish a baseline of the students’ knowledge, which was then repeated post-session to measure learning progress. Initially, the class provided a brief overview of computer vision, defining it, highlighting its goals, and mentioning prevalent libraries like OpenCV and Scikit-Image. Following this, the session delved into broader Artificial Intelligence (AI) topics, covering general concepts of Machine Learning (ML) and Deep Learning (DL), the primary ML algorithm types, and fundamental neural network concepts, including types of layers, convolution operations, and performance metrics. The discussion also discussed troubleshooting techniques like data augmentation, learning rate adjustments, and dataset bias correction. Subsequently, the lecture differentiated between two-stage and one-stage object detection algorithms, exploring their advantages and drawbacks and facilitating a conversation about choosing the appropriate method based on different scenarios.

In the practical application phase, instructions were given on dataset creation—either from publicly available sources or from scratch—including the labeling and data augmentation processes. The training was conducted using Google Colab, which offers free GPU usage and compatibility with the Darknet framework, primarily developed for Linux systems, thus enabling the training of older model types.

For the implementation demonstration, the use of OpenCV to load trained model weights and integrate with a Raspberry Pi 4-equipped video camera was showcased, enabling real-time object detection, just as Figure 11 demonstrates. The session concluded with a discussion on potential real-world applications of these algorithms, emphasizing the importance of translating the learned concepts to various fields.

### 3.1 Testing proof of concept

The testing of this proof of concept involved analyzing the impact of a short-notice class on student knowledge using ANOVA tests to detect any significant differences in test scores before and after the session. ANOVA (Analysis of Variance) was selected for this study to assess the statistical significance of differences between the groups’ average scores before and after the educational intervention. The choice of ANOVA is particularly apt for this scenario because it simultaneously compares the means of multiple groups, which is essential for cohesively analyzing the test scores from different student groups and time points. It provides a clear understanding of the variability within and between groups, distinguishing whether the changes in scores are due to intervention or random variations. ANOVA’s efficiency in handling complex experimental designs and larger datasets makes it a robust choice for studies, even those with smaller sample sizes like this proof of concept (St and Wold, 1989). Furthermore, it brings statistical rigor to the research by calculating a *p*-value, thereby facilitating a scientific evaluation of the null hypothesis against the alternative hypothesis. This methodological approach ensures that the investigation into the educational framework’s impact on student learning, particularly in complex subjects like computer vision and AI algorithms, is both comprehensive and statistically validated. Initially, 13 undergraduate engineering students from various majors—including mechatronics, mechanical, robotics, and computer science—participated. Their pre-class test average was 8.0, improving to 9.08 post-class. The one-way ANOVA test yielded a *p*-value of 0.046, suggesting a statistically significant improvement, even with the caveat of a small sample size. In a further attempt to validate the framework’s educational potential, another group of 25 computer science engineering undergraduates was exposed to the framework through documents detailing its industrial application, accompanied by Python programs and labeled images. The average grade before the course was 8.32; after that, it increased to 9.32. The ANOVA test shows a *p*-value of approximately 0.0000205, indicating a statistically significant improvement in the student’s grades after the course. Most students demonstrated a solid understanding of computer vision post-session, and about half grasped the YOLO algorithm and its implementation well. Student feedback was generally positive, highlighting the effectiveness of the course in conveying general concepts, though they expressed a desire for more structured guidance in algorithm implementation. The framework’s utility is underscored by these initial educational interventions, showing its potential to enhance understanding complex AI topics like computer vision and the YOLO algorithm. However, it is important to note that these tests serve only as preliminary indicators of the framework’s effectiveness in an educational setting. The results, while promising, point to the necessity for broader and more in-depth testing to establish the framework as a robust educational tool.

Table 1 delineates the questions directed towards the students, aimed at gauging their comprehension across various facets of the implementation process. Spanning from theoretical underpinnings to nuanced technical aspects and practical application inquiries, these three distinct sections are designed to assess the students’ grasp of the algorithm’s functionality and their ability to implement it effectively across diverse scenarios.

**TABLE 1:** Questions were posed to the students from three distinct perspectives.

Questions		
Theoretical concepts	Technical details	Implementation questions
What is computer vision?	What are anchor boxes, and why are they important in YOLO?	For which of the following tasks can YOLO be used?
What does YOLO stand for in the context of computer vision?	Which of the following is a core component of the YOLO architecture?	What type of data is crucial for training a YOLO model?
How does YOLO differ from traditional object detection methods?	In YOLO, what is meant by “non-max suppression”?	How can YOLO be optimized for real-time processing?
		What is a potential challenge when implementing YOLO for object detection in new domains?

The evaluation should be conducted on a large cohort of students enrolled in the same major and academic year. This approach ensures a homogeneous group with a similar level of knowledge and background. In addition to traditional examinations, students will be required to complete a project demonstrating their ability to apply the knowledge gained in the course. This project will practically assess their understanding of the tools discussed during the lectures.

The students’ projects will not only reveal their ability to implement theoretical knowledge in practical scenarios but will also allow them to evaluate the advantages and limitations of these tools. This critical assessment aims to prevent the misuse of complex solutions when simpler alternatives are adequate. Furthermore, successfully executing these projects can boost the students’ confidence in their ability to apply these methodologies in real-world settings, thereby contributing to the rapid evolution and adaptation of industry standards to innovative tools and concepts introduced in academia.

Finally, the students were asked to leave a comment about the syllabus and the perception of their change in understanding on this topic; most students thanked the syllabus for including general concepts and linking them to more specific things such as object detection, which is often left out in other teaching approaches, commenting that this helped them to understand more easily the topics covered.

## 4 Future work

The subsequent phase of the project entails a thorough analysis of the implementations executed by students within their respective simulated manufacturing environments. This analysis will focus on quantifying the system’s impact on various performance metrics, such as efficiency, accuracy, or any other relevant standard, to compare results from scenarios where the system was not utilized. This evaluative approach aims to provide concrete data on the practical benefits and potential limitations of incorporating object detection systems in industrial contexts.

Furthermore, the student’s comprehension and retention of the subject matter will be assessed through three examinations, strategically administered at different stages of the lesson plan. The first test is an initial gauge of the students’ baseline knowledge regarding object detection concepts, providing a reference point for subsequent assessments. The second examination follows the teacher’s presentation of the theoretical framework, aimed at measuring the students’ grasp of these abstract concepts and the educational impact of the academic instruction. The final assessment comes at the project’s conclusion, designed to evaluate the knowledge acquired throughout the course and explore students’ innovative thought processes. It is important to note that the questions across these three tests will differ, preventing students from merely memorizing answers and thus ensuring a more accurate measure of their genuine understanding and learning progression. However, the complexity and scope of the questions will be consistent, with some items prompting students to conceptualize practical applications of their learned skills within real-world manufacturing settings. This approach seeks to assess whether the educational goals have been met regarding subject matter comprehension and the student’s ability to envisage and appreciate the broader possibilities presented by this technology.

This should be done on a larger group of students from the same major and the same year to have a more uniform group of students with similar knowledge. Since previous knowledge may vary since some majors include general AI concepts on their syllabus, 30 students from different majors will be asked to take the course and perform the project. Apart from the exams, a project presented by the students will also be requested to see how they are implementing the knowledge acquired to present a working solution using the tools presented in the class. This project can showcase their understanding of the pros and cons of these tools to avoid using them unnecessarily when more straightforward solutions can be used; it will also help to build confidence in the student to implement this in their workplace in the future, helping the industry to evolve more quickly and to adapt the new tools proposed by the academic world.

Incorporating a targeted survey in the educational context aims to dissect and understand how different sections of a course contribute to student learning and conceptual comprehension. The primary intention behind this survey is to capture the students’ perceptions regarding how specific course elements facilitate their learning process. By meticulously questioning students about each course segment, educators can identify which aspects are most effective in enhancing understanding and where improvements are necessary. This systematic approach ensures that no critical feedback is overlooked, fostering a more comprehensive enhancement of the educational experience.

The design will be informed by preexisting research in the field of educational surveys to construct a robust and reliable survey. Previous studies and surveys serve as valuable blueprints, offering insights into structuring questions and framing objectives to yield meaningful data. While the core structure of these precedent surveys might be retained, modifications will be made to tailor the questions to the specific context and objectives of the current course under evaluation, as exemplified in reference (Vasan et al., 2009).

Moreover, as suggested in the reference (Sarkar et al., 2021), the survey will incorporate multiple-choice questions to streamline the response process and enable students to articulate their perceptions accurately. This format facilitates a more straightforward analysis of the collected data and accommodates students’ diverse viewpoints, allowing for a more complete understanding of their learning experiences. As Willems and Dreiling (2022) highlighted, student motivation is an essential factor to consider in the survey’s design. Motivation plays a critical role in the learning process, influencing both student engagement and perceptions. Understanding the motivational drivers can provide deeper insights into how course elements are perceived and interact with students’ intrinsic and extrinsic motivations, thereby shaping their educational experience.

This focused survey intends to unravel the specific impacts of course segments on student understanding, leveraging existing research to formulate a comprehensive and effective tool. By considering students’ perceptions and motivations, the survey aims to gauge each course element’s effectiveness and guide actionable improvements, enhancing the educational process for learners.

Below is a proposed survey that could be implemented in future studies to assess students’ learning outcomes and perceptions of the subject matter.

### 4.1 Topic understanding and perception survey

#### 4.1.1 Demographic information

1. What is your year of study?
  • Freshman
  • Sophomore
  • Junior
  • Senior
  • Graduate
2. What is your major/field of study?

#### 4.1.2 Course content evaluation

3. Please rate how well you understood the concepts presented in the following sections of the course (1 = Not at all, 5 = Extremely well):
  • Computer vision 1: _______
  • Classification algorithms 2: _______
  • Yolo algorithms 3: _______
  • Implementing Yolo algorithms 4: _______
4. What aspects helped you understand the material better for each section mentioned above? (Select all that apply)
  • Lectures
  • Textbook readings
  • Practical exercises
  • Group discussions
  • Online resources
  • Other (please specify)

## 5 learning experience

5. How motivated did you feel to engage with each topic section?
  • Computar vision: Very unmotivated - Very motivated
  • Classification algorithms 2: Very unmotivated - Very motivated
  • Yolo algorithms 3: Very unmotivated - Very motivated
  • Implementing Yolo Algorithms 4: Very unmotivated - Very motivated
6. What motivated you to engage with the topic content? (Select all that apply)
  • Interest in the subject
  • Course requirement
  • Instructor’s teaching style
  • Peer influence
  • Career prospects
  • Other (please specify)

## 6 Feedback and improvement

7. What challenges did you face in understanding the concepts of the topic?
8. How could these sections be improved to enhance your understanding and learning experience?
9. Is there anything else about the course that could be changed or improved to help you learn better?

## 7 Overall evaluation

Overall, how satisfied are you with the way the course helped you understand the subject matter? (1 = Not at all satisfied, 5 = Extremely satisfied).

This survey structure is proposed to elicit detailed feedback on the effectiveness of various course components, student motivations, and perceived challenges, which can be used to tailor and enhance the educational experience.

## 8 Conclusion

This paper has successfully designed a novel educational framework by integrating a V-model methodology with project-based learning. It is explicitly tailored to the intricacies of artificial intelligence (AI) and its application in computer vision (CV). This innovative approach demystifies AI technologies and showcases their broad potential across various research and industrial domains. Implementing this pedagogical model has been instrumental in deepening students’ comprehension of object detection algorithms while equipping them with valuable hands-on experience through projects that mirror real-world manufacturing scenarios. This immersive learning process enhances academic achievement and prepares students for the professional world, offering them a solid portfolio of practical implementations that could elevate their career opportunities. From an industrial perspective, the paper highlights the substantial benefits of integrating AI systems into operations, notably enhancing efficiency and safety. The proactive use of AI for quality control and the prevention of accidents, such as through detecting safety equipment misuse or machinery malfunctions, exemplifies the practical value of these technologies. These applications facilitate more efficient maintenance processes and contribute to the overall safety and productivity of the workplace. Furthermore, as revealed through this research, the scope of AI’s impact extends beyond immediate manufacturing processes to include broader operational aspects like routine maintenance, thus advocating for a comprehensive integration of AI across various operational spheres. This holistic application of AI underscores its significance in environments where procedural efficiency and safety are paramount. The contribution of this paper lies in its creation of a structured yet flexible educational framework that not only fosters a deep understanding of AI and CV technologies but also emphasizes their practical application in real-world scenarios. This approach advances academic knowledge and bridges the gap between theoretical learning and industrial application, thereby fostering a generation of well-equipped professionals to drive innovation and enhance efficiency in their respective fields. Through this framework, the study makes a compelling case for the transformative potential of AI, promoting a proactive embrace of these technologies in both educational and industrial settings.

## Data Availability

The raw data supporting the conclusion of this article will be made available by the authors, without undue reservation.
