# Supplementary material for: Learning manufacturing computer vision systems using tiny YOLOv4
Source: Front Robot AI. 2024 Jun 12;11:1331249. doi: 10.3389/frobt.2024.1331249 (PMC11199777; doi:10.3389/frobt.2024.1331249)
Supplement: Supplementary file 1 [file DataSheet1.docx]

Supplementary Material

Learning Manufacturing Computer Vision Systems Using

Tiny YOLO v4

Adan Medina^1^, Russel Bradley^2^, Wenhao Xu^2^, Pedro Ponce^3*^, Brian Anthony^2^, Arturo Molina^3^

^1^School of Engineering and Sciences, Tecnologico de Monterrey, Calle del Puente 222, Ejidos de Huipulco, Tlalpan, CDMX, Mexico, [adan.mr@tec.mx](mailto:adan.mr@tec.mx).

^2^School of Engineering, Department of Mechanical Engineering, Massachusetts Institute of Technology, Cambridge, Massachusetts, United States, [russelb@mit.edu](mailto:russelb@mit.edu), [wenhaoxu@mit.edu](mailto:wenhaoxu@mit.edu), [banthony@mit.edu](mailto:banthony@mit.edu).

^3^Institute of Advanced Materials and Sustainable Manufacturing, Tecnologico de Monterrey, Calle del Puente 222, Ejidos de Huipulco, Tlalpan, CDMX, Mexico, [pedro.ponce@tec.mx](mailto:pedro.ponce@tec.mx), [armolina@tec.mx](mailto:armolina@tec.mx).

*** Correspondence:**Corresponding Author
[pedro.ponce@tec.mx](mailto:pedro.ponce@tec.mx)

# Project’s Web Page Link

The most up to date supporting materials and additional content for teaching the content presented in the article can be found in the project web page below.

<https://fredfactory.mit.edu/post/learning-manufacturing-computer-vision-systems-using-tiny-yolov4>

# Dataset, Notebook and Code

The supporting materials used for the example presented in the paper in the version as of the publication date can be found in the link below. The repository contains the dataset, notebook for training the YOLOv4 model and the Python script for deploying the model in a Raspberry Pi microcomputer.

<https://zenodo.org/records/11204799>

**DOI:** 10.5281/zenodo.11204798
